# Central inhibition and placebo analgesia

**DOI:** 10.1186/1744-8069-1-21

**Published:** 2005-06-30

**Authors:** Min Zhuo

**Affiliations:** 1 Department of Physiology, Faculty of Medicine, University of Toronto, University of Toronto Centre for the Study of Pain, 1 King's College Circle, University of Toronto, Toronto, M5S 1A8, Canada

## 

Most neuroscientists measure neuronal activity in the brain to predict or explore the contribution of neurons to physiological/pathological functions of the brain. For example, if an increase in neuronal activity is detected in one brain region by peripheral noxious stimuli, we call this area the pain region or pain matrix. In the case of activity detected during conscious processes, such as placebo treatment, we label these regions as responsible for the placebo treatment. Due to limited access to the conscious brain and the reductionist nature of most modern neuroscience, we are all reluctant to explore more sophisticated hypotheses. Few studies have performed experiments at neuronal network levels.

Like other higher order brain functions, the investigation into the neuronal mechanisms of placebo analgesia is proving to be difficult for clinicians and basic researchers. For example, there are individual differences in the response to placebos, called responders and non-responders. Certain populations of individuals show a significantly greater response to placebos than others. Evidence for environmental and social impacts on brain development is increasing; therefore, individual differences in higher order brain functions such as placebo analgesia is expected. Levine and colleagues (1978) performed a classic study on placebo analgesia [1]. Their hypothesis is based on the discovery of endogenous opioid peptides in the central nervous system. Placebo analgesia in patients was blocked by naloxone, an opioid receptor antagonist. These experiments indicate that the endogenous analgesia systems are likely to be activated during placebo analgesia, possibly at different levels of the central nervous system.

## Major recent findings: alternations in ACC brain activity and placebo analgesia

Even though animal experiments are well designed and controlled, it is very difficult to test mechanisms of placebo analgesia in animal preparations. One major study using human brain imaging showed the activation of forebrain structures, in particular the ACC, during placebo analgesia. The correlation between ACC activity and placebo analgesia, as well as opioid analgesia, was shown in another study [2]. Considering the sensitivity of placebo analgesia to naloxone, it is proposed that increased ACC activity recruits endogenous analgesia systems by projecting innervations to the midbrain periaqueductal gray (PAG). Since a large number of opioid receptors are found in the ACC [3,4], opioids are likely to be released within the ACC (as measured by increased activity during imaging) during placebo analgesia [2]. Activation of these opioid receptors somehow activates endogenous analgesia systems originating from the PAG, thereby producing analgesic effects [5]. Although functional imaging data is interesting and important, the explanation of these findings is obscure due to the limitations of human imaging techniques. Here, I would like to propose that several brain mechanisms might contribute to placebo analgesia, which may not be revealed by human imaging studies.

## Activation of the ACC in the human brain

Neurons in the ACC receive inputs from highly wired networks (see [6]). Therefore, it is not surprising that activation of the ACC is reported during different physiological or pathological conditions. For example, ACC activity is increased by acute pain, opioid analgesia and placebo analgesia [2,7,8]. It is proposed that the ACC functions heterogeneously, playing various functions through its multiple connections with other cortical and subcortical nuclei. This heterogeneity indicates that ACC neurons are not solely responsible for physiological functions, such as pain, attention etc, and this complexity certainly makes it more difficult for scientists to obtain simple and easily interpreted results (Table 1).

## Opposite types of neurotransmission in the brain: excitatory/inhibitory

It is well documented that neurons are highly connected by chemical synapses in the brain and spinal cord [9]. Excitatory synaptic transmission is mainly carried out by glutamate. In both the spinal dorsal horn and ACC, fast excitatory transmission is mediated by glutamate [3,10,11]. To prevent and control widely distributed excitatory transmission, neurons have local inhibitory synapses to prevent them from over-excitation and undergoing glutamate-induced neuronal death. Therefore, at any given moment, neurons that form excitatory and inhibitory synapses are likely to be continuously active. For this reason, it is critical to remember that measured neuronal activity may contain both excitatory and inhibitory neuronal activities, and that some of the measured activity may not play a physiological function.

## Limitations of imaging and recording studies in monkeys and humans

Human imaging and awake monkey recordings provide physiological evidence for the role of neurons in performing a single, or a series, of functions. Under well controlled experimental conditions, ACC neurons show consistent patterns of activity [12,13]. However, due to poor access to individual neurons in experimental subjects, many key questions remain unanswered. First, in most monkey studies neither the exact location nor the type of neuron (pyramidal versus local interneurons) recorded is known. In the case of human brain imaging studies, it is not very clear whether the increase in metabolic activity during testing is due to changes in neurons versus glia cells, inhibitory versus excitatory neurons (although, in most cases, investigators interpret this activity as excitatory neuronal activity), or local modulatory neurons containing neuropeptides. All of these factors limit our ability to compare discoveries from conscious monkeys or humans to the findings of molecular and cellular studies using rats and mice. Furthermore, we may still miss sub-threshold activity or activity in other key structures using human imaging studies. It is necessary to keep in mind that a big change may not necessarily be an important one or one directly related to the task.

## Do ACC neurons serve as a higher cortical activator of the endogenous analgesia system (PAG-RVM-spinal cord)?

After discussing central neural transmission and the limitations of imaging studies, we are ready to explore one hypothesis proposed for placebo analgesia. That is, activation of the ACC triggers the endogenous analgesia system and modulates sensory transmission at the level of the spinal cord by descending inhibitory mechanisms, thereby contributing to placebo analgesia (see Discussion in [2]). Evidence for such descending inhibitory modulatory systems comes from electrophysiological, pharmacological, and behavioral studies in animals. For example, activation of the PAG or RVM by electrical stimulation or glutamate decreases responses of spinal cord dorsal horn neurons, including those that send ascending projections, to peripheral noxious stimulation (see [5,14]) and inhibits the spinal nociceptive tail-flick reflex. Anatomical studies reveal that neurons in the dorsal horn receive descending inhibitory innervations. Application of a pharmacological antagonist can block theses inhibitory effects. This key hypothesis assumes that the connections from the ACC to the midbrain/brainstem trigger these descending inhibitory systems. However, recent studies show that activation of ACC neurons by electrical stimulation, or the activation of glutamate synapses, does not produce any analgesic effects [15-19]. In contrast, facilitation of the spinal nociceptive tail-flick reflex, aversive learning or fear memory was reliably induced by ACC stimulation [20]. Consistent with this notion, inhibition of opioid receptors in the ACC inhibited excitatory synaptic transmission [3,4], reduced nociceptive hot-plate responses, inhibited behavioral nociceptive responses to formalin injection and blocked chronic pain in some clinical studies [21,22]. Furthermore, enhanced excitatory synaptic transmission and long-term plasticity were found in ACC neurons after tissue injury [23]. Intracellular recordings from ACC pyramidal neurons reveal that injury causes rapid excitatory responses in the ACC. Studies using genetically modified mice lend further support to the excitatory role of the ACC in chronic pain. Genetic over-expression of NMDA NR2B receptors selectively enhanced chronic pain [24], and genetic deletion of calcium-stimulated adenylyl cyclases (AC) 1 and 8 blocked persistent pain in various pain models [25]. In summary, enhanced excitatory transmission in the ACC is unlikely to activate endogenous analgesia systems.

## ACC activity during placebo analgesia

An alternative explanation for the role of ACC activation in placebo analgesia is increased activity of local inhibitory neurons. Increased inhibitory activity within the ACC thus reduces the amount of excitatory responses to painful stimuli. By doing so, subjects report less pain (placebo analgesia). Based on the network connections that the ACC has with other pain-related neuronal structures, we propose that ACC activation can lead to placebo analgesia through the following neuronal mechanisms:

Mechanism I. Inhibition of pain-processing neurons in the ACC

**Figure 1:**
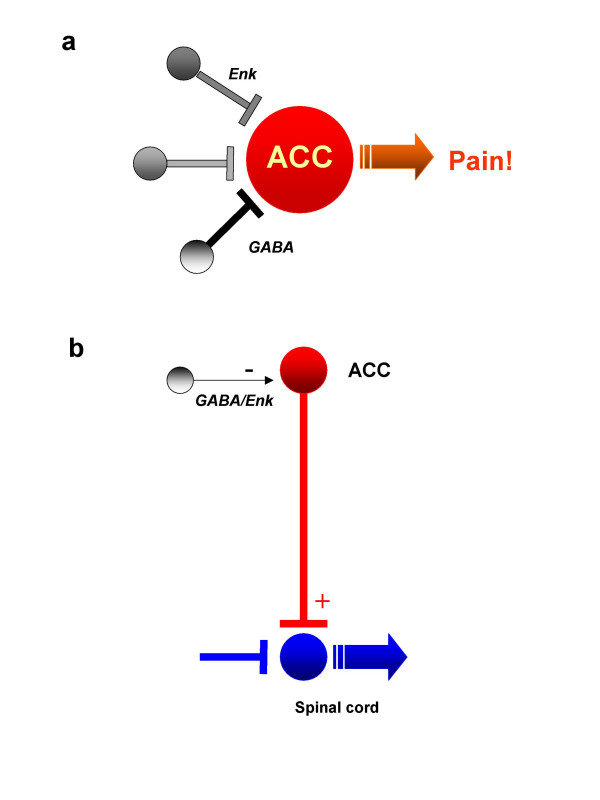
**A model neuronal network explaining placebo analgesia-related activation of ACC neurons. **Placebo leads to activation of inhibitory neurons within the ACC. These inhibitory neurons then release an inhibitory neurotransmitter, GABA. GABA acts on postsynaptic GABA receptors to inhibit ACC neurons that are involved in pain perception. In some neurons, endogenous neuropeptides such as enkaphalin (Enk) may also be released to produce similar inhibitory effects (a). Inhibitory neurons may also affect ACC neurons that form descending facilitatory innervations with the spinal cord dorsal horn. Activation of inhibitory neurons within the ACC causes the reduction of descending facilitatory influences. The reduced facilitatory influence on spinal nociceptive transmission therefore produces analgesic effects.

Many neurons in the ACC respond to acute pain and the amount of this activation is related to pain unpleasantness. Activation of inhibitory neurons in the ACC can affect the excitability of these neurons by releasing GABA onto their postsynaptic receptors. Consequently, the excitability of ACC neurons is reduced, and neurons respond less to noxious stimuli (Figure 1a).

Mechanism II. Activation of local opioid-containing neurons in the ACC

Similar to Mechanism I, neurons containing opioid peptides may be activated. Opioids may act presynaptically and/or postsynaptically to inhibit excitatory synaptic transmission and reduce neuronal responses to subsequent peripheral noxious stimuli. This mechanism could explain the fact that some placebo effects are sensitive to blockade by naloxone (Figure 1a).

Mechanism III. Inhibition of descending facilitatory modulation from the ACC

The release of the inhibitory neurotransmitter GABA and/or opioids will reduce the excitability of ACC neurons that send descending innervations directly or indirectly to RVM neurons. Consequently, descending facilitatory influences will be reduced (Figure 1b).

*Mechanism IV. Mixed activation of excitatory and inhibitory transmission by placebo treatment with the net result within the ACC being reduced excitatory transmission*.

## Conclusion and future directions

Recent progress in neuroscience research provides us new opportunities to investigate higher order brain functions such as placebo analgesia. However, the mechanism is likely to be complex. Long-term plasticity of both excitatory and inhibitory transmission, postsynaptic trafficking and recycling of various receptors, activation of immediate early genes, and constant changes in synaptic structure and connections, are potential mechanisms for higher brain functions. Integrative approaches, from genetically manipulated mice to human brain imaging, will improve our understanding of placebo analgesia.

**Table 1:** Evidence for the pro-nociceptive effects of ACC

**ACC manipulation**	Nociceptive (+)/analgesia (-)
Anatomic lesions in the ACC	-
Electrical stimulation locally in the ACC	+
Chemical activation within the ACC	+
Opioid injection in the ACC	-
Electrophysiological recordings from the ACC	+
Imaging data	+/-
